# The Capacity to Be Alone Moderates Psychopathological Symptoms and Social Networks Use in Adolescents during the COVID-19 Pandemic

**DOI:** 10.3390/ijerph182111033

**Published:** 2021-10-20

**Authors:** Silvia Cimino, Luca Cerniglia

**Affiliations:** 1 Department of Dynamic, Clinical, and Health Psychology, University of Rome, 00186 Rome, Italy; silvia.cimino@uniroma1.it; 2 Faculty of Psychology, International Telematic University Uninettuno, 00186 Rome, Italy

**Keywords:** adolescence, COVID-19, social networks sites, capacity to be alone, psychopathology

## Abstract

During the COVID-19 pandemic, adolescents could not leave their house freely, meet up with friends, or attend school; previous literature showed that youths under enforced confinement or quarantine were five times more likely to suffer from psychopathological symptoms and use social networks sites (SNs) greatly. This study aimed to verify whether the quality of the parent-adolescent relationship could predict youths’ psychopathological symptoms and their SN use during the pandemic, and to evaluate the possible moderator role of their the capacity to be alone. Seven hundred and thirty-nine (*n* = 739) adolescents were recruited from the general population during the COVID-19 lockdown in Italy, and they were administered The Capacity to be Alone Scale, The BSMAS, the YSR, and the Perceived Filial Self-efficacy Scale. Our results confirmed a direct effect of the perceived filial self-efficacy on the psychopathological symptoms so that a poorer perceived quality of the relationship with the caregivers predicted higher psychopathological symptoms in youths. Moreover, greater social networks use was predictive of psychopathological symptoms in adolescents. Our results also showed a significant interaction effect between adolescents’ perceived filial efficacy and the capacity to be alone on SN use and on psychopathological symptoms. These results suggest that youths’ response to the confinement during the pandemic is influenced both by individual characteristics (the capacity to be alone) and by relational variables (the perceived filial self-efficacy).

## 1. Introduction

The Sars-Cov-2 virus has been spreading throughout the world since February 2020, causing more than four million deaths and impacting people’s interpersonal and social interactions, freedom of movement and travel, as well as work, school, and family habits [1]. In response to the COVID-19 pandemic, governments have implemented disease containment measures such as school closures, social distancing, and home confinement. Youths experienced separation from their classmates, instructors, extended families, and community networks for long periods of time [2]. Although lockdown periods and quarantines have mostly been documented to be associated with psychopathological symptoms in adults [3], recent literature proposed these difficulties to be especially problematic for adolescents, probably due to the particular importance of the peer group for identification and support in this period of development [4,5]. It has been noted that the COVID-19 pandemic can be defined as a potentially traumatic environmental experience challenging individuals’ resilience capacities to cope with distress, uncertainty and subversion of previous and consolidated habits [6]; however, only a few studies have so far specifically investigated the psychological outcomes of the pandemic in adolescents experiencing solitude from their peers and every-day familiar environments (schools, gyms, etc.) during lockdowns and/or quarantines (see for example [7,8,9]). It must be acknowledged that in most cases adolescents had not been completely isolated and alone during the pandemic because they could nonetheless count on their parents’ and/or relatives’ presence. Although adolescents predominantly use peers as references and support, in times of extreme difficulty, youths (especially during the first years of adolescence) might return to their family as a source of comfort, security, and emotional regulation. However, as Goodman and Gotlib have posited, the quality of the relationship between caregivers and adolescents may vary [10], and if the quality of the relationship between youths and their caregivers is low and the environmental context within which the youth lives is not sensitive and positive, affect regulation processes in offspring may be hindered. Thus, youths could not find support in the family and may experience a number of psychopathological symptoms both in the internalizing and externalizing areas [11]. The capacity to be alone, which is one of the most important indicators of emotional maturity [12], has been shown to be a significant buffer that may help individuals cope with the negative impacts of risk variables and distressing situations [13] and to moderate the effect of the low quality of parent-youths’ relationship on adolescents’ psychopathology [14]. The capacity to be alone has also been shown to mitigate the effects of social media overuse on psychological distress in adolescents [15]. Individuals with a high capacity to be alone, who are usually characterized by adaptive emotion regulation processes, seem to be more able to cope with the adverse consequences of problematic use of social network sites (SNs); conversely, those with low capacity to be alone are suggested to suffer more depressive symptoms, anxiety and negative emotional states related to high use of SN, due to a reduced ability to regulating their emotional states. Given the importance of this capacity, however, no study (apart from [16]) has so far focused on the potential (null, positive or negative) effect of this variable on the psychopathological outcomes of adolescents during the pandemic.

Adolescents are renowned for using social media to connect with peers (e.g. Instagram, TikTok) and several studies have shown that youths turn to social network sites to cope with negativity [17]. The COVID-19 lockdowns brought both distress (high emotional activation) and boredom (sense of void and confinement with emotional deactivation) in the life of adolescents and cut out their interactions with peers. As both hyperarousal and hypo-arousal are unpleasant degrees of activation and social interactions are central in adolescents’ lives, they may have used SN during the lockdowns to regulate their inner states to reach an adaptive equilibrium and to maintain exchanges with peers. Importantly, previous literature not focused on the pandemic period has shown that the overuse of SN may be associated with a number of psychopathological symptoms in adolescence [18]. However, no study, to the best of our knowledge, has investigated the effect of massive SN use by adolescents during the COVID-19 pandemic. In fact, in a condition where adolescents were forced to stay indoors and have SN as the only form of communication with peers, it could be posited that frequent and prolonged use of social network sites cannot be defined as excessive and potentially problematic. However, it has been demonstrated that the potentially problematic effect of the intense use of social networks is not only linked to the motivation driving adolescents to stay online, so that although the motive to use SN could be considered as adaptive (keep the contacts with their peers), the use itself of such technology could lead to negative outcomes. Indeed, high use of SN may cause over-stimulation, with screen-time putting the nervous system into fight-or-flight mode, causing dysregulation, disorganization, and distress [19]. Moreover, previous literature has not addressed the role of the capacity to be alone in moderating the effect of social networks use on adolescents’ psychological distress during this period. In this study we hypothesized that the perceived quality of the relationship with their caregivers predicted social networks sites use and psychopathological symptoms in adolescents. Moreover, we hypothesized that the capacity to be alone played a moderating role.

## 2. Materials and Methods

### 2.1. Sample, Recruitment, and Procedure

Seven hundred and thirty-nine (*n* = 739) adolescents from the general population (M_age_ = 13.4; SD = 1.2; 51% males) were contacted through social network ads during the COVID-19 lockdown in Italy (from March to May 2020). A consecutive sampling method was used to form a convenience sample, and the inclusion criteria were: (1) no referred psychiatric diagnosis in the subjects and/or in their parents; (2) no medical condition present in the subjects at the moment of the recruitment; (3) no medical and/or psychological treatment pursued; and (4) no COVID-19 contagion in any member of the family and no death of any close relative associated with COVID-19. All recruited adolescents were students. All contacted subjects agreed to participate in this study and their parents or guardian signed the written informant consent, consistent with the Declaration of Helsinki. Before its start, the present study was authorized by the Ethical Committee of Sapienza (N. 0000809-2020). All measures (described below) were administered via an online platform and adolescents filled out all questionnaires remotely. Our study was therefore consistent with the indications of the Horizon Programme 2020 (H2020) that recommended focusing on assessment, prevention and intervention, also via technology-mediated tools and with the COVID-19-related guidelines suggesting interpersonal distancing and remote-administered research.

### 2.2. Measures

*The Capacity to be Alone scale* [20] is made up of two inter-correlated l0-item Likert subscales (solitary coping scale and solitary comfort scale) ranging from 1 (never) to 4 (always). This scale is concerned with the specific use of confinement to deal with stress (e.g., “Being alone is not healing for me”). The solitary comfort scale assesses an individual’s emotional comfort or discomfort while alone (for example, “I can’t have pleasure until I’m with someone”). In the present study we chose to use a unidimensional score with higher scores indicating higher capacity to be alone. Cronbach’s alpha for the entire scale in this study was 0.85. The reliability coefficients for solitary coping and solitary comfort were 0.75 and 0.76, respectively.

*The Bergen Social Media Addiction Scale* (BSMAS) [21] evaluates experiences in the use of social media within a 12-month period. It comprises six items rated on a 5-point Likert scale (from 1 = Very rarely to 5 = Very often) and is related to core addiction elements (salience, mood modification, tolerance, withdrawal, conflict, and relapse). The items of this measure usually refer to the last year (e.g., “How often during the last year have you used social media so much that it has had a negative impact on your job/studies?” and “How often during the last year have you felt an urge to use social media more and more?”). In this study, the items were modified to tap the three-month period from March 2020 to May 2020. Higher scores in this scale indicate higher problematic use of SN. The clinical cut-off for this measure is 24 [22]. As in previous studies on the general population [23], in this research, no subject exceeded the clinical cut-off for problematic use and the internal consistency of the scale was good. In this study the Cronbach’s alpha was 0.80.

*The Youth self-report* (YSR) [24] is a self-report questionnaire that covers behavioral and emotional problems. It contains 112 problem items, which are scored on a three-point scale (0 = not true, 1 = somewhat or sometimes true, 2 = very or often true). The YSR total problem scale can be divided into nine syndrome subscales: Withdrawn, Somatic complaints, Anxious/depressed, Social problems, Thought problems, Attention problems, Delinquent behavior, Aggressive behavior, Self-destruct Identity. Withdrawn, Somatic complaints and Anxious/Depressed together comprise a broad ‘‘Internalizing” dimension (31 items), whereas Delinquent and Aggressive behaviors together constitute an ‘‘Externalizing” dimension (32 items). Higher scores on these scales indicate more maladaptive functioning. Some YSR items are included in the ‘‘Other problems” subscale (32 items). For the purpose of this study we only used the Total Problem score, which taps all the symptoms and is a measure of perceived general maladjustment level. In this study Cronbach’s alphas was 0.81.

*The perceived filial self-efficacy* questionnaire measures perceived filial self-efficacy, i.e., youths’ perceptions of their parents’ accessibility, sensitivity, and support in everyday settings as well as in a hypothetical important point in their lives. Higher scores on this tool suggest that parents are regarded to be more supportive [25]. Adolescents’ perceived filial self-efficacy (PFSE) was assessed using 16 questions ranging from “strongly disagree” (ranked as 1) to “strongly agree” (scoring as 7) on a seven -point scale, assessing belief in their ability to discuss personal concerns with their parents even under challenging conditions; nurture positive affective relationships and control negative emotional reactions to them; persuade parents to see their point of view on controversial topics; manage stress caused by parental marital problems; and impact parental views and social behaviors in a favourable way. ‘‘I can persuade my parents to see my point of view when it varies from theirs” is a metric for efficacy in dealing with potentially difficult topics. The measure was developed using knowledge of prototypical scenarios that teenagers face with their parents [26]. In this study, the Cronbach’s alpha was 0.87.

### 2.3. Statistical Analyses

Gender and age were included as control variables in this study since previous research indicated that they were strongly connected to the key variables in this study [27].

First, we examined the means, standard deviations, and bivariate correlations for all research variables using descriptive statistics and Pearson correlations. Second, we utilized Hayes’ suggested SPSS (IBM SPSS, Version 24.0. Armonk, NY, USA) macro PROCESS (model 8) to evaluate the proposed moderated mediation model [28]. This SPSS macro was used to evaluate mediating and moderating models in numerous studies, and it demonstrated greater statistical testability [29].

## 3. Results

All of the observed variables’ means, standard deviations, and correlations are listed in Table 1. Social network site use was shown to be negatively linked with the perceived filial self-efficacy and the capacity to be alone; SN use was moreover positively correlated with psychopathological symptoms. The capacity to be alone was linked to a lower level of psychopathological symptoms. Gender and age showed no significant correlation with all of the study variables.

The suggested moderated mediation model effect was tested using Hayes’ [28] SPSS macro PROCESS. The major findings are given in Table 2. The total effect model (*F* (1,738) = 48.12, *R*^2^ = 0.45, *p* < 0.001), the mediator variable model (*F* (1,738) = 22.15, *R*^2^ = 0.35, *p* < 0.001), and the dependent variable model (*F*(1,738) = 34.23, *R*^2^ = 0.41, *p* < 0.001) were all significant after controlling for adolescents’ gender and age. Social network use (*β* = 0.22, *p* < 0.001) and perceived filial efficacy (*β* = 0.25, *p* < 0.001) were shown to be (respectively positively and negatively) associated with psychopathological symptoms. The Sobel test was used to evaluate the relevance of the indirect effect of perceived filial efficacy on psychopathological symptoms via SN use. The findings revealed that SN use significantly moderated the association between perceived filial efficacy and psychopathological symptoms (z = 3.63, *p* < 0.001).

Interaction effects were investigated using the PROCESS macro (Model 8) [28]. There was a significant interaction effect between perceived filial efficacy and the capacity to be alone on SN use in the mediator variable model (*B* = 0.24, *p* < 0.001). There was a significant perceived filial efficacy x capacity to be alone interaction impact on psychopathological symptoms in the dependent variable model (*B* = 0.16, *p* < 0.05). The ability to be alone affected both the relationship between perceived filial efficacy and psychological difficulties as well as the link between perceived filial efficacy and SN use.

## 4. Discussion

The restrictions used to contain the COVID-19 pandemic have resulted in a prolonged period of stress for adults, adolescents, and children [30]. It has been posited that adolescents could use social networks to keep in touch with peers and relatives, and rely on the support of parents and caregivers to cope with these psychological difficulties [31,32]. It has also been suggested that the capacity to be alone is a protection factor from the psychological distress caused by the pandemic [27]. However, to our knowledge, no studies have investigated the links between all of these variables (the quality of parent-adolescent relationship as perceived by the adolescent, SN use, the capacity to be alone, and psychopathological symptoms) in youths during COVID-19 lockdowns.

This study aimed to fill this gap in literature and proposed that the quality of parent-adolescent relationships is proposed as the predictor of the psychopathological symptoms and SN use, and the capacity to be alone is suggested as a moderator. Our results confirmed a direct effect of the perceived filial self-efficacy on the psychopathological symptoms so that a poorer perceived quality of the relationship with the caregivers predicted higher psychopathological symptoms in youths. This was an expected result, given the large bulk of literature that had already demonstrated how the low perceived quality of caregiving was a strong predictor of the psychological difficulties in children and adolescents [33,34]. The added value of this study, however, is that no other research had yet shown this effect in adolescents during the COVID-19 pandemic and the conditions of social confinement in which the effects of the quality of the relationship on psychological distress were tested. Therefore, although several previous studies focused on this effect, investigations during these unique times were necessary to confirm former research. 

Moreover, our results showed that greater social network use was predictive of psychopathological symptoms in adolescents. Numerous previous studies have demonstrated that SN overuse can be associated with psychopathology in adolescents [35,36]. On the other hand, with specific regard to the lockdowns, some authors have posited that the use of social networks constituted a protective factor from the development of psychological difficulties in adolescents during the forced confinement, as they allowed contacts with peers and relatives [37]. In our sample this protective effect has not proven to be present; on the contrary, in our study SN use fostered psychological problems in youths. Notably, no subject exceeded the clinical cut-off for problematic use of the SN at the BSMAS questionnaire; therefore, one cannot assume that psychopathological symptoms were predicted by problematic use of the social networks. Rather, our results showed that significant (yet not clinically problematic) use of SN by adolescents during the lockdown was predictive of higher psychopathological risk. This result has potential important implications for the implementation of social policies for the management of possible future lockdowns because it suggests that encouraging the use of SN during times of social confinement is not necessarily positive and could (partially counter intuitively) lead to poorer psychological well-being in youths. The mechanism undergoing this effect remains unclear and further studies should be performed to disentangle it. However, we can speculate that while during everyday life SN constitutes a valid means for keeping in touch with peers and allows them to get into contact with other adolescents because it is also supported by physical encounters in shared physical environments [38], the imposed social confinement during the lockdown impeded these exchanges and hindered the supportive effect of face-to-face meetings. This hypothesis is potentially confirmed by the further result of our study that showed that SN use in adolescents significantly moderated the association between perceived filial efficacy and psychopathological symptoms. In our sample, adolescents who perceived less sensitivity and support from their parents and used social networks more intensely were more likely to develop more serious psychopathological symptoms. It can be hypothesized that those youths who do not perceive to find emotional comfort from their caregivers during the lockdowns used SN to find support and/or cope with negative emotions. But social network use seems to fail in this task and, on the contrary, fosters more severe symptoms. It is widely known that psychopathology can be prevented by the protective effect of strong relationships (both with parents and with peers), which are effective at buffering distress, leading to better social and psychological outcomes [39]. But strong bonds are usually supported by physical proximity [40]. If social confinement is protracted in time (as in the case of the lockdowns during the pandemic), even those relationships continuing online after starting face-to-face may weaken, therefore diminishing their protective effect towards psychological suffering, and paradoxically adding distress and frustration to adolescents’ experience, eventually leading to higher symptoms.

Moreover, our results showed a significant interaction effect between adolescents’ perceived filial efficacy and the capacity to be alone on SN use and on psychopathological symptoms. In sum, adolescents with (perceived) supportive and sensitive parents who also could rely on their capacity to be alone were less likely to suffer significant psychological distress and used SN in a less problematic fashion. These results confirm previous research which posited how the capacity to be alone may make adolescents more able to handle risk factors and distressing situations [16], especially when they can rely on supportive and sensitive caregivers [41,42]. Being capable of coping with negative emotions, we can speculate that these adolescents were less likely to use social networks as a means of affective regulation, therefore using them in a less problematic way.

This study has limitations. First, all used measures were self-reported questionnaires that could suffer social desirability, and it might have been informative to have an objective assessment administered by professional psychologists. Second, we could not assess possible parental psychopathology, whereas caregivers’ psychopathological risk is widely recognized as one of the main risk factors for negative outcomes in children and adolescents. In fact, family has a key role in the intergenerational transmission of psychopathological behavior [43,44]. Third, although we proposed a predictive model to describe the links between the study variables, this was a non-longitudinal study, and the interpretation of the effects remains exclusively speculative. Finally, the size of the study was relatively small, considering that it is a community sample and that lockdowns during the COVID-19 pandemic affected millions of adolescents.

## 5. Conclusions

The results of this study suggest that youths’ response to the confinement during the pandemic in terms of psychopathological symptoms and social network use can be influenced both by individual characteristics (the capacity to be alone) and by relational variables (perceived filial self-efficacy). In fact, adolescents with (perceived) supportive and sensitive parents who also could rely on their ability to be alone were less likely to suffer high psychological distress and used SN in a less problematic fashion.

## Figures and Tables

**Table 1:** Descriptive statistics and correlations among all of the study variables.

Variables	M	SD	1	2	3	4	5	6
1. Gender	1.54	0.35	1					
2. Age	13.43	0.82	0.043	1				
3. BSMAS	2.34	0.79	0.036	0.024	1			
4. PFSE	4.02	1.21	0.051	0.032	−0.091 *	1		
5. YSR	40.3	13.1	0.062	0.042	0.233 **	0.062	1	
6. Capacity to be alone	2.45	1.21	0.034	0.021	−0.098 *	0.057	−0.219 **	1

Note. BSMAS = Bergen Social Media Addiction Scale; PFSE = Perceived Filial Self-Efficacy; YSR = Youth Self Report; *p* < 0.01 **, *p* < 0.05 *.

**Table 2:** Regression and moderated mediation results.

Model							
***Model 1: Total effect model***							
***R***	***R*** **^2^**	***F***	***p***	***B***	***SE***	***t***	***p***
0.45	0.23	48.12	<0.001				
Constant				0.075	0.08	1.15	>0.05
Gender				0.049	0.04	1.49	>0.05
PFSE				0.256 ***	0.03	10.21	<0.001
***Model 2: Mediator variable model***							
***R***	***R*** **^2^**	***F***	***p***	***B***	***SE***	***t***	***p***
0.35	0.21	22.15	<0.001	0.01			
Constant				0.03	0.07	31.23	<0.01
Gender				0.02	0.08	0.31	>0.05
Capacity to be alone				0.16 ***	0.05	2.67	<0.001
PFSE X Capacity to be alone				0.24 ***	0.07	3.65	<0.001
***Model 3: Dependent variable model***							
***R***	***R*** **^2^**	***F***	***p***	***B***	***SE***	***t***	***p***
0.41	0.26	34.23	<0.001				
Constant				0.01	0.08	4.21	<0.01
Gender				0.04	0.07	1.21	>0.05
BSMAS				0.22 ***	0.05	1.12	<0.001
PFSE				−0.25 ***	0.07	8.45	<0.001
Capacity to be alone				−0.17 ***	0.08	2.65	<0.001
PFSE X Capacity to be alone				−0.16 ***	0.07	−2.43	<0.001

Note. Unstandardized regression coefficients are reported; *p* < 0.001 ***.

## Data Availability

The data presented in this study are openly available in FigShare at doi:10.6084/m9.figshare.14402444.
